# A review of heat stress in chickens. Part I: Insights into physiology and gut health

**DOI:** 10.3389/fphys.2022.934381

**Published:** 2022-08-04

**Authors:** Giorgio Brugaletta, Jean-Rémi Teyssier, Samuel J. Rochell, Sami Dridi, Federico Sirri

**Affiliations:** ^1^ Department of Agricultural and Food Sciences, Alma Mater Studiorum—University of Bologna, Bologna, Italy; ^2^ Center of Excellence for Poultry Science, University of Arkansas, Fayetteville, AR, United States

**Keywords:** chicken, heat stress, physiology, metabolism, gut health

## Abstract

Heat stress (HS) compromises the yield and quality of poultry products and endangers the sustainability of the poultry industry. Despite being homeothermic, chickens, especially fast-growing broiler lines, are particularly sensitive to HS due to the phylogenetic absence of sweat glands, along with the artificial selection-caused increase in metabolic rates and limited development of cardiovascular and respiratory systems. Clinical signs and consequences of HS are multifaceted and include alterations in behavior (e.g., lethargy, decreased feed intake, and panting), metabolism (e.g., catabolic state, fat accumulation, and reduced skeletal muscle accretion), general homeostasis (e.g., alkalosis, hormonal imbalance, immunodeficiency, inflammation, and oxidative stress), and gastrointestinal tract function (e.g., digestive and absorptive disorders, enteritis, paracellular barrier failure, and dysbiosis). Poultry scientists and companies have made great efforts to develop effective solutions to counteract the detrimental effects of HS on health and performance of chickens. Feeding and nutrition have been shown to play a key role in combating HS in chicken husbandry. Nutritional strategies that enhance protein and energy utilization as well as dietary interventions intended to restore intestinal eubiosis are of increasing interest because of the marked effects of HS on feed intake, nutrient metabolism, and gut health. Hence, the present review series, divided into Part I and Part II, seeks to synthesize information on the effects of HS on physiology, gut health, and performance of chickens, with emphasis on potential solutions adopted in broiler chicken nutrition to alleviate these effects. Part I provides introductory knowledge on HS physiology to make good use of the nutritional themes covered by Part II.

## Introduction

Heat stress (HS) affects performance, health, and welfare of commercially-reared birds (Renaudeau et al., 2012; Rostagno, 2020) and alters their meat (Song and King, 2015; Wang et al., 2017; Zaboli et al., 2019) and egg quality (Mack et al., 2013; Barrett et al., 2019), thereby endangering the sustainability of the poultry industry. This environmental stressor has commonly impacted poultry flocks raised in tropical and subtropical regions of the world. However, as a result of global warming, high environmental temperatures have become a large-scale issue that severely threatens poultry producers located in temperate areas as well (Renaudeau et al., 2012). Extreme heatwaves have already caused devastating events for the poultry industry, such as the sudden death of more than 700,000 birds in California (Nienaber and Hahn, 2007). St-Pierre et al. (2003) reported that the financial burden of HS amounts to $128–165 million per year for the US poultry industry alone. Although this figure still represents a general reference, recent estimates cannot be easily found in the literature.

In addition to causing evident changes in chicken behavior (Wang W. C. et al., 2018), HS negatively acts upon metabolism and general homeostasis (Rhoads et al., 2013) and impairs the functionality of the digestive system (Rostagno, 2020). Interestingly, several physiological and pathophysiological responses to HS are evolutionary conserved across different animal species (Lambert et al., 2002; Pearce et al., 2012; Koch et al., 2019; Kaufman et al., 2021), including humans (Snipe et al., 2018). For instance, the reduction in feed intake is one of the most common HS reactions because it is an effective way to limit the generation of metabolic heat due to digestion, absorption, and nutrient metabolism (Baumgard and Rhoads, 2013).

Given its tremendous relevance to the whole sector, poultry scientists and companies have been committed to developing reliable tools against HS. Engineering solutions and equipment intended to optimize the environmental control of poultry houses, along with management measures and genetic selection, can undoubtedly aid poultry producers in counteracting hot conditions (Lin et al., 2006b; Naga Raja Kumari and Narendra Nath, 2018; Saeed et al., 2019; Liang et al., 2020; Wasti et al., 2020; Goel, 2021; Nawaz et al., 2021; Vandana et al., 2021). Feeding strategies and dietary interventions can also help relieve HS effects on poultry (Gous and Morris, 2005; Lin et al., 2006b; Wasti et al., 2020; Vandana et al., 2021). For instance, increasing energy and nutrient density of the diet can counterbalance the decreased feed consumption of birds exposed to HS (Wasti et al., 2020). Moreover, researchers dealing with HS have been testing feed supplements aimed at promoting gastrointestinal (GI) health (Lian et al., 2020), which is a mainstay for the modern animal science and livestock industry (Kogut and Arsenault, 2016). Gut health is multifaceted and simultaneously influenced by composition and properties of the diet, digestion and absorption processes, integrity of the GI epithelium, plasticity and resilience of the GI immune system, and dynamics of the GI microbiota (Brugaletta et al., 2020). Pioneering studies conducted in the 1980s revealed that the GI microbiota is a main target of HS and that dietary supplementation of probiotic blends can attenuate HS in chickens (Suzuki et al., 1983, 1989). Probiotics have been shown to drive the formation of a desirable and protective GI microbiota (Baldwin et al., 2018), while properly reestablishing eubiosis following environmental stress, such as the exposure to elevated temperatures (Sugiharto et al., 2017). Along with probiotics, other GI microbiota modulators and potentially gut health-enhancing additives have been tested in poultry nutrition to promote eubiosis under HS conditions, such as prebiotics, synbiotics, postbiotics, phytochemicals, and amino acids, to name but a few (Lian et al., 2020; He et al., 2021).

Over the last years, HS-mediated alterations of physiology and gut health of chickens have received considerable attention. Therefore, this review series composed of Part I and Part II, was conceived to summarize relevant knowledge about these topics and examine some feeding and nutritional interventions that have been proposed to mitigate HS in broiler chickens. The present Part I discusses the effects of HS on physiology and gut health of chickens, while Part II (Teyssier et al., 2022a) provides an overview of potential solutions employed in broiler chicken nutrition to minimize the detrimental effects of HS.

## Heat stress effects on physiology of chickens

Chickens are homeotherms that can keep their body temperature tightly regulated across a wide range of external temperatures. However, high environmental temperatures can overwhelm the thermoregulatory mechanisms, causing an imbalance between the amount of metabolic heat produced by chickens and their own capacity to dissipate body heat to the environment. This alteration results in an abnormal increase in body temperature (hyperthermia) and triggers HS (Renaudeau et al., 2012; Rostagno, 2020). In addition to being potentially lethal, HS has a broad-spectrum effect on behavior, physiology, gut health, welfare, and productive performance of chickens. It is worth pointing out that fast-growing and highly efficient broiler chickens (Havenstein et al., 1994, 2003b; Zuidhof et al., 2014; Tallentire et al., 2018), the outcome of about 70 years of genetic progress, are even less thermotolerant and more susceptible to HS than slow-growing lines due to extremely high metabolic rates and poorly developed cardiovascular and respiratory systems (Cahaner and Leenstra, 1992; Yunis and Cahaner, 1999; Havenstein et al., 2003a; Gous and Morris, 2005; Lu et al., 2007; Yahav, 2009; Xu et al., 2018). In this regard, Gogoi et al. (2021) recently proved that the physiological response to HS is more severe and heat tolerance is lower in heavy broilers than in lighter birds of the same line and age.

### Heat stress and behavior

“Cooling behaviors” (Wang W. C. et al., 2018) are the most prominent HS-caused modifications in chicken behavior. As their name suggests, these abnormal behaviors are intended to cool the body down to restore normothermia. Chickens lack sweat glands, which would facilitate latent heat loss by evaporation of the perspiration, and have relatively limited unfeathered body surface areas to provide an effective loss of sensible heat through conduction, radiation, and convection (Nichelmann et al., 1986; Yunis and Cahaner, 1999; Renaudeau et al., 2012; Rostagno, 2020). As the ambient temperature rises, the thermal gradient between the body surface and the surrounding environment lessens while the dissipation of sensible heat becomes decreasingly effective. Therefore, chickens suffering from environment-induced hyperthermia increase their respiratory rate (thermal tachypnea/polypnea or panting) to maximize the loss of latent heat *via* evaporation of water from the respiratory tract (Jukes, 1971; Teeter et al., 1985). While sensible heat loss is restricted by the body-to-environment thermal gradient, relative humidity imposes a ceiling on water evaporation and, therefore, on latent heat dissipation (Renaudeau et al., 2012). Thus, elevated ambient temperature associated with high relative humidity considerably limit heat removal from the body and magnify the injurious effects of HS on chickens (Rajaei-Sharifabadi et al., 2017; Goel, 2021). Under persistent HS conditions, thermal polypnea turns into a slower and deeper panting phase, also called thermal hyperpnea (Hales, 1973; IUPS Thermal Commission, 1987; Renaudeau et al., 2012). Even though panting improves evaporative cooling through latent heat dissipation, it has some drawbacks for chickens (Marder and Arad, 1989). Dehydration, the most intuitive panting-related disadvantage, usually results in higher water requirement and consumption (Wang W. C. et al., 2018). Panting also increases CO_2_ exhalation leading to hypocapnia and, eventually, to respiratory alkalosis, a disorder of the acid-base balance (Richards, 1970; Marder and Arad, 1989; Renaudeau et al., 2012; Beckford et al., 2020; Wasti et al., 2020). Alkalosis poses a risk to the egg industry because it reduces blood ionized calcium and therefore negatively affects eggshell mineralization (Odom et al., 1986). However, HS-induced respiratory alkalosis is a great threat to broiler growers as well (Teeter et al., 1985; Borges et al., 2007). Chickens subjected to HS frequently lift their wings (Wang W. C. et al., 2018) to expose body areas uncovered by feathers in an attempt to enhance the sensible heat flow toward the environment. Despite being fundamental to preserving or reestablishing euthermia, panting and raising wings are energy-intensive activities (Brackenbury and Avery, 1980; Dale and Fuller, 1980) which deplete the amount of calories that would be allocated to productive purposes (Yahav et al., 2004; Baumgard and Rhoads, 2013).

Chickens kept at high temperatures become lethargic, spending more time resting (e.g., squatting close to the ground) and less time feeding and walking. This unfavorably affects feed intake (Wang W. C. et al., 2018) and skeletal health (Hester et al., 2013). Limiting feed consumption is a highly conserved survival mechanism employed by animals to reduce the thermogenesis from digestive, absorptive, and nutrient utilization processes (Baumgard and Rhoads, 2013). Reduced performance of heat-stressed chickens have traditionally been attributed to reduced feed intake (Dale and Fuller, 1980; Teeter et al., 1985). However, pair-feeding models—adopted to minimize the confounding effects of dissimilar feed consumption between birds under thermoneutral conditions and their heat-stressed counterparts—revealed that up to 40% of body weight gain loss of broilers subjected to HS are unrelated to anorexia (Dale and Fuller, 1980; Geraert et al., 1996a; Ain Baziz et al., 1996; Lu et al., 2007; Zuo et al., 2015; Lu et al., 2018; Teyssier et al., 2022b). Readers are referred to Part II of this review series for more information on the effects of HS on feed intake (Teyssier et al., 2022a). Table 1 provides an overview of heat stress effects on chicken behavior.

**TABLE 1:** Overview of heat stress effects on chicken behavior.

Class	Heat stress effect	Pros	Cons	References^a^
Behavior	↑ respiratory rate (thermal polypnea or panting) → thermal hyperpnea	↑ latent heat dissipation (evaporative heat loss through the respiratory tract)	Dehydration → higher water requirement and consumption	**Richards (1970)**, **Jukes (1971)**, **Brackenbury and Avery (1980)**, **Dale and Fuller (1980)**, **Teeter et al. (1985)**, **Odom et al. (1986)**, **Marder and Arad (1989)**, **Yahav et al. (2004)**, **Borges et al. (2007)**, Renaudeau et al. (2012), Rhoads et al. (2013), **Wang W. C. et al. (2018)**, **Beckford et al. (2020)**, **Wasti et al. (2020**)
			↑ CO_2_ loss → hypocapnia → respiratory alkalosis (acid-base imbalance) → ↓ blood calcium for eggshell mineralization and ↓ growth performance	
			↑ energy expenditure to maintain euthermia → ↓ performance	
	Wing lifting	↑ exposition of unfeathered body surfaces → ↑ sensible heat loss	↑ energy expenditure to maintain euthermia → ↓ performance	**Dale and Fuller (1980)**, Baumgard and Rhoads (2013), **Wang W. C. et al. (2018)**
	Lethargy → ↓ feeding and walking	↓ metabolic heat from digestion, absorption, and nutrient utilization	↓ performance	**Dale and Fuller (1980)**, **Teeter et al. (1985)**, **Geraert et al. (1996a)**, Baumgard and Rhoads (2013), **Hester et al. (2013)**, **Wang W. C. et al. (2018)**
			↓ bone/skeletal health	

Upward arrow (↑), increase; downward arrow (↓), decrease; rightward arrow (→), consequence/degeneration.

a Include studies on non-avian species that have exhibited comparable heat stress effects and responses to those observed in chickens. Studies with the focus on chickens or poultry are highlighted in bold.

### Heat stress and lipid metabolism

Direct effects of HS upon physiology, other than reduced feed intake, remarkably contribute to impair chicken performance (Dale and Fuller, 1980; Geraert et al., 1996a; Renaudeau et al., 2012). Heat-stressed animals paradoxically show a restricted fat mobilization notwithstanding their negative energy balance and catabolic state (Baumgard and Rhoads, 2013). Indeed, not only chickens (Bobek et al., 1997; Lu et al., 2018), but also pigs (Pearce et al., 2013a; Victoria Sanz Fernandez et al., 2015), and dairy cattle (Rhoads et al., 2009) kept in warm environments show a progressive reduction in circulating non-esterified (free) fatty acids (NEFA)—a reliable indicator of lipid metabolism—suggesting a limited use of fat energy stores. Extensive research has also revealed that heat-stressed chickens deposit more visceral (abdominal), subcutaneous, and intramuscular fat (Kleiber and Dougherty, 1934; Kubena et al., 1972; Ain Baziz et al., 1996; Yunianto et al., 1997; He et al., 2015; Lu et al., 2018, 2019). A greater lipid retention at the peripheral body sites can further hinder the dissipation of sensible heat (Renaudeau et al., 2012), increasing the risk of severe hyperthermia. The hampered fat mobilization is a metabolic adaptation likely due to hyperinsulinemia triggered by HS, at least in pigs and cattle (Baumgard and Rhoads, 2013). In contrast to mammals, however, heat-stressed chickens do not usually show a spike in blood insulin levels (Geraert et al., 1996b; Tang et al., 2013; Belhadj Slimen et al., 2016), although Lu et al. (2019) reported an increase and a decrease in circulating insulin at 7 and 14 days of HS, respectively. Moreover, avian insulin lacks a powerful antilipolytic effect, while the importance of its signaling cascades in the adipose tissue of chickens is still unclear and a matter of debate (Dupont et al., 2012, 2015). Therefore, several questions about the role of insulin in fat metabolism of chickens undergoing HS remain unanswered at present. It is worth noting, however, that the altered lipid metabolism is not limited to a reduced utilization of fat storages because heat-challenged chickens also show an overexpression of proteins involved in the hepatic *de novo* lipogenesis, along with fat accumulation in the liver (Flees et al., 2017; Lu et al., 2019). HS effects on chicken lipid metabolism are summarized in Table 2.

**TABLE 2:** Overview of heat stress effects on chicken lipid metabolism.

Class	Heat stress effect	Pros	Cons	References^a^
Lipid metabolism	↓ fat mobilization and ↑ hepatic lipogenesis → ↑ fat retention and deposition	—	↑ carcass adiposity	**Kleiber and Dougherty (1934)**, **Kubena et al. (1972)**, **Ain Baziz et al. (1996)**, **Geraert et al. (1996a)**, **Yunianto et al. (1997)**, **Bobek et al. (1997)**, Rhoads et al. (2009), Renaudeau et al. (2012), Baumgard and Rhoads (2013), Pearce et al. (2013a), Victoria Sanz Fernandez et al. (2015), **He et al. (2015)**, **Flees et al. (2017)**, **Lu et al. (2018)** **,** **Lu et al. (2019**)
			↓ sensible heat dissipation	

Upward arrow (↑), increase; downward arrow (↓), decrease; rightward arrow (→), consequence/degeneration.

a Include studies on non-avian species that have exhibited comparable heat stress effects and responses to those observed in chickens. Studies with the focus on chickens or poultry are highlighted in bold.

### Heat stress and skeletal muscle protein metabolism

In addition to an increase in fat content, HS has been demonstrated to alter the carcass composition of broiler chickens by lowering the lean tissue proportion, especially the breast yield (Howlider and Rose, 1989; Geraert et al., 1996a; Ain Baziz et al., 1996; Temim et al., 2000; Zuo et al., 2015; Lu et al., 2018; Qaid and Al-Garadi, 2021; Zampiga et al., 2021). First molecular insights suggested that HS-mediated depression in muscle protein deposition is mostly attributable to a reduced protein synthesis rather than a more pronounced protein breakdown (Temim et al., 2000). Zuo et al. (2015) showed, however, that the cause for the diminished lean mass accretion can be muscle-specific, with the breast showing a decreased protein synthesis while the thigh an augmented protein degradation. They also associated the impaired protein synthesis with a lower expression of insulin-like growth factor 1 (IGF-1), phosphatidylinositol 3-kinase (PI3K), and p70S6 kinase (S6K) and the higher protein degradation with an upregulation of muscle atrophy F-box (MAFbx or atrogin-1). Ma et al. (2021) recently confirmed the modifications in S6K and MAFbx expression caused by HS. S6K is indispensable in controlling protein synthesis and muscle development in chickens (Bigot et al., 2003; Duchêne et al., 2008; Everaert et al., 2010). Interestingly, Boussaid-Om Ezzine et al. (2010) detected a limited response of the S6K signaling pathway to anabolic stimuli in heat-stressed broiler chickens. Lu et al. (2018) measured an increase in blood uric acid, urea, and proteinogenic amino acids (AA)—in spite of a marked decrease in feed intake and breast yield—along with a reduction in glucose and NEFA. Consequently, they postulated that heat-exposed chickens mobilize protein reservoirs of skeletal muscles, particularly the breast, to compensate for the inability to extract energy from stored fat. In this regard, plasmatic levels of creatine, 3-methylhistidine, and urea have been used as biomarkers to assess muscle protein breakdown induced by HS (Rhoads et al., 2013). The hypothesis formulated by Lu et al. (2018) has been supported by Ma et al. (2021) who found that HS reduces plasmatic glucogenic AA, increases AA uptake of the liver and its glucogenic potential, and enhances the activity of hepatic transaminases that deaminize AA to make them precursors for gluconeogenesis. Furthermore, Zampiga et al. (2021) observed that heat-stressed broilers exhibit a drop in blood glucogenic precursors and breast muscle free AA, despite a rise in circulating protein-building AA concomitant with a substantial feed intake reduction. Table 3 presents a summary of heat stress effects on skeletal muscle protein metabolism of chickens.

**TABLE 3:** Overview of heat stress effects on skeletal muscle protein metabolism of chickens.

Class	Heat stress effect	Pros	Cons	References^a^
Protein metabolism	↓ protein synthesis and ↑ protein breakdown in skeletal muscles	Supply of glucogenic precursors to the liver	↓ lean tissue yield (especially breast yield)	**Howlider and Rose (1989)**, **Ain Baziz et al. (1996)**, **Geraert et al. (1996a)** **,** **Temim et al. (2000)** **,** **Boussaid-Om Ezzine et al. (2010)** **,** Rhoads et al. (2013), **Zuo et al. (2015)** **,** **Lu et al. (2018)** **,** **Ma et al. (2021)** **,** **Qaid and Al-Garadi (2021)** **,** **Zampiga et al. (2021)**

Upward arrow (↑), increase; downward arrow (↓), decrease; rightward arrow (→), consequence/degeneration.

a Include studies on non-avian species that have exhibited comparable heat stress effects and responses to those observed in chickens. Studies with the focus on chickens or poultry are highlighted in bold.

### Heat stress and hormonal levels

Chickens subjected to HS share numerous hormonal variations with mammalian species. HS activates the hypothalamic-pituitary-adrenal axis, leading to a marked increase in circulating glucocorticoids, particularly corticosterone (Geraert et al., 1996b; Yunianto et al., 1997; Quinteiro-Filho et al., 2010, 2012; Rajaei-Sharifabadi et al., 2017; Lu et al., 2019; Beckford et al., 2020; Ma et al., 2021). In chickens, high levels of corticosterone have been reported to decrease growth potential, induce proteolysis and suppress protein synthesis in skeletal muscles, and increase fat deposition (Decuypere and Buyse, 1988; Dong et al., 2007; Yuan et al., 2008), all of which are typical HS consequences (Rhoads et al., 2013). It has been proposed that corticosterone impairs muscle protein metabolism by inducing the abovementioned changes in S6K and MAFbx expression (Ma et al., 2021), while also exerting a lipogenic effect by promoting the expression of fatty acid synthase (FASN) in hepatocytes and adipocytes (Gonzalez-Rivas et al., 2020). However, a recent investigation demonstrated that treating heat-stressed chicken myotubes with corticosterone does not intensify proteolysis and does not increase the expression of MAFbx compared to the HS treatment alone (Furukawa et al., 2021). The latter interesting results have been obtained *in vitro*, and therefore further research may be needed to elucidate the role of corticosterone in the altered protein metabolism of heat-stressed chickens.

Additionally, since hypercorticosteronemia is immunosuppressive (Quinteiro-Filho et al., 2010), heat-challenged chickens have a compromised immunocompetence and are more prone to infectious diseases (Renaudeau et al., 2012; Farag and Alagawany, 2018; Chauhan et al., 2021). In this regard, Hirakawa et al. (2020) detected serious immunological disorders in heat-stressed broilers, such as a decrease in immunoglobulin production against a prototype antigen as well as atrophy and dysfunction of primary and secondary lymphoid tissues accompanied by lymphocyte depression. The authors mentioned hypercorticosteronemia among the plausible reasons for these anomalies in the immune system. Corticosterone-related immune dysfunctions of chickens have thoroughly been described by Shini et al. (2010).

Reductions in hematic triiodothyronine (T_3_) and thyroxine (T_4_) have also been observed in laying hens (de Andrade et al., 1977; Bobek et al., 1997) and broiler chickens (Geraert et al., 1996b; Yunianto et al., 1997; Sohail et al., 2010; Rajaei-Sharifabadi et al., 2017; Beckford et al., 2020) undergoing HS. These variations, which might be caused by decreased size and activity of the thyroid (Huston and Carmon, 1962; Dale and Fuller, 1980; Yunianto et al., 1997), have also been measured in heat-stressed dairy cattle (Chen et al., 2018). It has commonly been assumed that the thyroid response to high environmental temperatures is an adaptive mechanism that allows animals to lower their basal metabolism and thermogenesis in order to prevent overheating (Renaudeau et al., 2012; Chen et al., 2018; Gonzalez-Rivas et al., 2020). The hypothyroid-like condition can partly justify growth depression (McNabb and Darras, 2015), increased carcass adiposity (Decuypere and Buyse, 1988; Geraert et al., 1996b), and decreased egg production and shell quality (de Andrade et al., 1977) observed during HS. Table 4 briefly illustrates the effects of heat stress on chicken hormonal levels.

**TABLE 4:** Overview of heat stress effects on chicken hormonal levels.

Class	Heat stress effect	Pros	Cons	References^a^
Hormonal levels	Hypothalamic-pituitary-adrenal axis activation → ↑ circulating glucocorticoids (e.g., corticosterone)	—	↓ growth potential	**Decuypere and Buyse (1988)**, **Geraert et al. (1996b)**, **Yunianto et al. (1997)**, **Dong et al. (2007)**, **Yuan et al. (2008)**, **Quinteiro-Filho et al. (2010)** **,** **Quinteiro-Filho et al. (2012)**, Renaudeau et al. (2012), Rhoads et al. (2013), **Rajaei-Sharifabadi et al. (2017)**, **Lu et al. (2019)**, **Beckford et al. (2020)**, Gonzalez-Rivas et al. (2020), **Hirakawa et al. (2020)**, **Wasti et al. (2020)**, Chauhan et al. (2021), **Ma et al. (2021**)
			↓ protein synthesis and ↑ protein breakdown in skeletal muscles → ↓ lean tissue yield	
			↑ fat deposition	
			↓ immunocompetence → ↑ infectious susceptibility and health care costs	
			↓ GI barrier	
	Hypothyroid-like state	↓ basal metabolism and thermogenesis → ↓ metabolic heat generation	↓ growth potential	**Huston and Carmon (1962)**, **de Andrade et al. (1977)**, **Dale and Fuller (1980)**, **Decuypere and Buyse (1988)**, **Geraert et al. (1996b)**, **Yunianto et al. (1997)**, **Sohail et al. (2010)**, Renaudeau et al. (2012), **McNabb and Darras (2015)**; **Rajaei-Sharifabadi et al. (2017)**, Chen et al. (2018), **Beckford et al. (2020)**, Gonzalez-Rivas et al. (2020)
			↑ carcass adiposity	
					↓ egg production and eggshell quality

Upward arrow (↑), increase; downward arrow (↓), decrease; rightward arrow (→), consequence/degeneration.

a Include studies on non-avian species that have exhibited comparable heat stress effects and responses to those observed in chickens. Studies with the focus on chickens or poultry are highlighted in bold.

## Heat stress effects on gut health of chickens

Gut health should be addressed in a holistic way (Oviedo-Rondón, 2019) by taking into consideration the major elements that synergistically affect it, namely the GI epithelium, the GI immune system, and the GI microbiota (Kogut et al., 2017). Being the largest body surface exposed to the environment, the gastrointestinal tract (GIT) is repeatedly threatened by a wide variety of harmful factors (Yegani and Korver, 2008), like noxious feed-derived compounds and pathogenic microorganisms. Applegate and Troche (2014) emphasized that the GIT accomplishes conflicting tasks, that is maximizing nutrient uptake while recognizing multiple antigenic stimuli and tolerating the resident microbiota. Hence, integrity and proper morpho-functionality of the GIT are of utmost importance in ensuring optimal health and productivity for chickens.

### Heat stress and gastrointestinal epithelium

The GI epithelium, arranged in a single-cell layer, takes an active part in the integrated gut immune system, forming a barrier reinforced by tight junction (TJ) proteins, secreting mucus and antimicrobial/host defense peptides (AMP/HDP), and expressing pattern recognition receptors (PRR) that orchestrate the enteral immune response (Smith et al., 2014; Chen et al., 2015; Broom and Kogut, 2018a).

TJs, the uppermost component of the apical junctional complex, seal the interstice between adjoining columnar epithelial cells (Farquhar and Palade, 1963) and encompass transmembrane (claudins and occludin) and scaffolding/peripheral/plaque proteins (zonula occludens—ZO). Through their binding domains, ZO anchor to claudins and occludin on one side and to the perijunctional actomyosin ring on the other side, thereby making a bridge between transmembrane TJs and the cytoskeleton (Ulluwishewa et al., 2011). TJs are primarily responsible for controlling the paracellular pathway that, unlike the pump- and channel-dependent transcellular transports, allows a passive transepithelial diffusion *via* two main routes, known as the pore pathway and the leak pathway (Dokladny et al., 2016). The pore pathway relies on claudins and limits the passage of charged and large molecules (greater than 4 Å), while the leak pathway, governed by occludin and ZO, can be crossed by big solutes, including bacterial lipopolysaccharides (LPS) (Anderson and Van Itallie, 2009; Dokladny et al., 2016; France and Turner, 2017). Under HS, the cardiovascular system responds in another evolutionary-preserved adaptation whereby a large volume of blood is shunted from the splanchnic tissues to peripheral areas of the body to maximize the dissipation of sensible heat (Hales, 1973; Lambert, 2009). This occurs to the detriment of the GIT because the altered blood pressure is mostly compensated by a sympathetically driven vasoconstriction of viscera (Table 5). The resulting hypoperfusion implicates a reduced supply of nutrients and oxygen to the GIT, which prompts deleterious effects on the intestinal mucosa (Lambert, 2009; Baumgard and Rhoads, 2013; Rostagno, 2020). In light of the remarkable energy and protein demands of the digestive system, a sub-optimal trophism of the GI epithelium negatively affects cell turnover and the maintenance of a robust intestinal barrier (Koutsos and Arias, 2006). On the other hand, the inadequate oxygenation leads to hypoxia, a condition that profoundly alters the cellular bioenergetic pathways and promotes the generation of reactive oxygen and nitrogen species (ROS and RNS, respectively) (Hall et al., 1999). Moreover, hyperthermia triggers ROS and RNS production per se (Hall et al., 2001) and impairs the enzymatic antioxidant systems (Farag and Alagawany, 2018), directly contributing to the establishment of a pro-oxidative scenario. Lin et al. (2006a) proved that elevated ambient temperatures provoke oxidative stress in chickens, while Tan et al. (2010) suggested that HS can depress the mitochondrial respiratory chain activity with consequent overproduction of ROS and oxidative injury. Worthy of mention is also the research on oxidative damage affecting the skeletal muscles, particularly the *Pectoralis major*, of heat-stressed broilers. Several authors demonstrated a rise in mitochondrial membrane potential, a high production of mitochondrial superoxide and ROS, and a considerable increase in malondialdehyde level (marker of lipid peroxidation) in breast muscles of broilers exposed to HS (Mujahid et al., 2006; Wang et al., 2009; Azad et al., 2010; Kikusato and Toyomizu, 2013). On the other hand, studies focused on the GIT have reported that oxidative stress destabilizes the TJ-regulated paracellular barrier and increases intestinal permeability (Rao, 2008; Bischoff et al., 2014). Myosin light-chain kinase (MLCK) is involved in this cascade of events because it regulates the circumferential contractions of the actomyosin ring and, indirectly, the TJ-controlled paracellular pathway (France and Turner, 2017). The actomyosin ring contractions can be triggered by several physiological and pathological stimuli. Oxidative stress has been shown to cause such contractions and, consequently, to affect the MLCK-regulated localization of ZO proteins and downregulate their expression, contributing to the deterioration of the paracellular barrier (González-Mariscal et al., 2011).

**TABLE 5:** Overview of heat stress effects on the cardiovascular system of chickens.

Class	Heat stress effect	Pros	Cons	References^a^
Cardiovascular system	Peripheral vasodilatation and GIT vasoconstriction	↑ sensible heat dissipation	GIT hypoperfusion →	Hales (1973), Hall et al. (1999), **Koutsos and Arias (2006)** **,** Rao (2008), Lambert (2009), Baumgard and Rhoads (2013), Bischoff et al. (2014), **Rostagno (2020)**
			↓ nutrient supply to the GIT → ↓ GI barrier and functionality	
			GIT hypoxia → oxidative stress → ↓ GI barrier and functionality	

Upward arrow (↑), increase; downward arrow (↓), decrease; rightward arrow (→), consequence/degeneration.

a Include studies on non-avian species that have exhibited comparable heat stress effects and responses to those observed in chickens. Studies with the focus on chickens or poultry are highlighted in bold.

Along with oxidative stress, the aforementioned increment in corticosterone levels further weakens the intestinal barrier (Quinteiro-Filho et al., 2012). Transepithelial electrical resistance (TEER)—i.e., the epithelium resistance to ion passage—and mucosa-to-serosa flux of marker probes (e.g., fluorescein isothiocyanate-dextran) have commonly been used to evaluate the paracellular barrier stability and integrity (Shen et al., 2011; Bischoff et al., 2014; Awad et al., 2017; Ma et al., 2018; Gilani et al., 2021). A steady paracellular pathway shows high values of TEER and effectively obstructs the flux of markers, whereas low TEER and high marker passage indicate poor barrier functions. HS has been shown to considerably reduce TEER and significantly increase the migration of marker tracers across the intestinal epithelium in numerous animal models (Dokladny et al., 2016), pigs (Pearce et al., 2013b), and broiler chickens (Song et al., 2014; Tabler et al., 2020). These variations are indicators of a “leaky gut” that barely holds noxious luminal compounds (Shen et al., 2011; Awad et al., 2017; Ma et al., 2018; Ruff et al., 2020). Translocation of pathogen-associated molecular patterns (PAMP) from the intestinal lumen to the underlying lamina propria is a major consequence of an increased paracellular permeability. The gut contains a massive amount of PAMPs, mainly LPS of Gram-negative bacteria (Wassenaar and Zimmermann, 2018), which can bind to a class of PRRs known as Toll-like receptors (TLR). Intestinal TLRs are particularly differentiated in chickens (Keestra et al., 2013) and have been shown to play a pivotal role in maintaining gut homeostasis and evoking inflammatory responses in the case of infections or other insults, such as hypoxia and tissue injury (Gribar et al., 2008). These receptors are also involved in epithelial cell proliferation, wound healing, stability of TJs, and modulation of immunoglobulin A (IgA) production and AMP expression (Abreu, 2010; Iizuka and Konno, 2011). Furthermore, TLRs are rather non-responsive to the multitude of commensal microorganisms inhabiting the GIT, yet are constantly responsive to PAMPs and host indicators of cell damage (Harris et al., 2006; Kogut et al., 2017; Madsen and Park, 2017). The ability to distinguish between useful microbes and those undesirable—or that can become such, like pathobionts (Round and Mazmanian, 2009)—is one of the most fascinating properties of the GI immune system (Mowat, 2018). At the basolateral membrane of the intestinal epithelium, LPS are recognized by the TLR4–MD-2 receptor complex (Shimazu et al., 1999; Abreu, 2010; Keestra et al., 2013) whose activation initiates an intracellular signaling cascade upregulating the expression of several pro-inflammatory cytokines (Vaure and Liu, 2014). The latter signaling molecules, also released by LPS-stimulated innate immune cells, foster a vicious cycle that deteriorates the intestinal barrier (Lambert, 2009). Tumor necrosis factor alpha (TNF-α), interleukin 1 beta (IL-1β), and interferon gamma (IFN-γ) have been reported to ruin the paracellular barrier, thereby increasing LPS leakage (Turner et al., 2014; Dokladny et al., 2016; Awad et al., 2017; Ma et al., 2018). Specifically, TNF-α has been shown to initiate the actomyosin ring contractions and, subsequently, to cause occludin internalization and TJ disassociation (Turner et al., 2014). Pro-inflammatory cytokines evoke a local inflammatory response aggravating the damages to the enteric mucosa. Quinteiro-Filho et al. (2010, 2012) reported that heat-stressed broilers manifest mild multifocal enteritis. Enteral inflammation has been shown to shorten the lifespan of enterocytes and cause crypt hyperplasia and villus atrophy (Smith et al., 2014). These alterations in intestinal epithelium morphology (microarchitecture), along with increased cell apoptosis and reduced cell proliferation, have recently been observed in broiler chickens exposed to HS (He et al., 2018a, 2018b; Liu et al., 2020, 2022; Nanto-Hara et al., 2020). In their pair-feeding study with broilers, Nanto-Hara et al. (2020) evidenced that intestinal morphological damage and increased intestinal permeability are direct consequences of HS rather than of feed intake reduction induced by HS itself. The resultant nutrient malabsorption and energy expenditure to sustain the GI immune response severely impact chicken performance and can be a predisposing factor for additional health problems (Broom and Kogut, 2018b).

In addition to initiating a local inflammation, luminal LPS can permeate the portal circulation whereby they reach and compromise the liver (Wang et al., 2015). Once exceeding the hepatic detoxification potential, LPS can diffuse throughout the bloodstream causing endotoxemia (Baumgard and Rhoads, 2013; Alhenaky et al., 2017; Epstein and Yanovich, 2019; Nanto-Hara et al., 2020). The resulting systemic inflammatory reactions force the organism to adjust nutrient partition and divert energy to support the immune system; this substantially depresses chicken performance (Broom and Kogut, 2018b; Ruff et al., 2020). At the worst, endotoxemia can lead to multi-organ failure and lethal septic shock (Wassenaar and Zimmermann, 2018).

According to Rostagno (2020), anomalies in the transcellular transport are another reason for intestinal permeability problems of chickens under HS. Indeed, a loss of epithelial integrity can degenerate into cell damage and consequent opening of TJ-independent pathways (France and Turner, 2017). Enteric bacteria can cross the altered and more permeable intestinal epithelium and, eventually, reach the liver or even migrate to other organs or tissues. For example, heat-stressed broilers showed a greater hepatic *Salmonella* invasion due to increased intestinal permeability (Alhenaky et al., 2017). This event, commonly called “bacterial translocation”, can be prelude to extraintestinal issues, such as deteriorations of liver functionality and health (Ilan, 2012; Ducatelle et al., 2018) as well as bacterial chondronecrosis with osteomyelitis (BCO) (Wideman, 2016). HS effects on the GI epithelium of chickens are summed up in Table 6.

**TABLE 6:** Overview of heat stress effects on the GI epithelium of chickens.

Class	Heat stress effect	Pros	Cons	References^a^
GI epithelium	Altered GI epithelium morphology (microarchitecture) and enterocyte lifecycle	—	Digestive and absorptive dysfunctions → ↓ performance	Lambert (2009), **Song et al. (2014)**, Vaure and Liu (2014), Wang et al. (2015), **Wideman (2016)**, **Dokladny et al. (2016)**, **Alhenaky et al. (2017)**, Awad et al. (2017), France and Turner (2017), Ma et al. (2018), Wassenaar and Zimmermann (2018), **Ducatelle et al. (2018)**, **He et al. (2018a)** **,** **He et al. (2018b)**, Epstein and Yanovich (2019), **Nanto-Hara et al. (2020)**, **Ruff et al. (2020** **)**, **Tabler et al. (2020)**, **Liu et al. (2020)** **,** **Liu et al. (2022**)
	↑ paracellular permeability (↓ transepithelial electrical resistance and ↑ mucosa-to-serosa flux of markers) → “leaky gut”			
	↓ GI epithelium integrity		LPS/endotoxins leakage →↑ pro-inflammatory cytokines → GI inflammation and ↓ GI barrier	
			↓ liver health and functionalityEndotoxemia → systemic inflammation, multi-organ failure, and septic shock	
			“Bacterial translocation” →↓ liver health and functionalitybacterial chondronecrosis with osteomyelitis	

Upward arrow (↑), increase; downward arrow (↓), decrease; rightward arrow (→), consequence/degeneration.

a Include studies on non-avian species that have exhibited comparable heat stress effects and responses to those observed in chickens. Studies with the focus on chickens or poultry are highlighted in bold.

### Heat stress and gastrointestinal microbiota

Microbiota and microbiome are similar-sounding words that are often used interchangeably. However, the microbiota represents a cluster of microorganisms residing in a specific environment (Marchesi and Ravel, 2015), such as an area of human or animal bodies (Clavijo and Flórez, 2018), while the microbiome unifies the metagenome of a microbiota (i.e., the collection of microbial genomes) and its surrounding environment (Marchesi and Ravel, 2015). The alimentary canal of chickens harbors an extremely complex microbial population that consists of bacteria, archea, protozoa, fungi, and viruses (Yeoman et al., 2012). The GI microbiota extends the genome of the host and substantially influences its physiology (Koutsos and Arias, 2006), almost acting as a supplementary—or “neglected” (Bocci, 1992)—organ. It is a widely held view that the GI microbiota is instrumental in programming and modulating both the gastroenteric (Iyer and Blumberg, 2018; Cheng et al., 2019) and systemic (Belkaid and Hand, 2014; Zheng et al., 2020) immune system of humans and animals, including poultry (Broom and Kogut, 2018c). This notion is supported by gnotobiotic models in which germ-free mice (Round and Mazmanian, 2009; Belkaid and Hand, 2014; Iyer and Blumberg, 2018) and chickens (Dibner et al., 2008) have been reported to suffer from severe developmental deficiencies and dysfunctions of the GI immunity. In addition to its immunogenic and immunoregulatory roles, the GI microbiota considerably influences growth, morphology, and function of the intestine in chickens (Dibner et al., 2008; Pan and Yu, 2014).

A myriad of host- and environment-related variables affects the GI microbiota (Kers et al., 2018). For instance, data from several studies indicate that high ambient temperatures can dramatically shape the GI microbiota. Specifically, it has been demonstrated that HS perturbs the GI microbiota in rats (Suzuki et al., 1983, 1989), poultry (Suzuki et al., 1983, 1989; Lan et al., 2004; Burkholder et al., 2008; Song et al., 2014; Wang X. J. et al., 2018; He et al., 2019b; Shi et al., 2019; Xing et al., 2019; Zhu et al., 2019; Liu et al., 2020; Wang et al., 2020; He et al., 2021; Liu et al., 2022), dairy cattle (Chen et al., 2018), and pigs (He et al., 2019a; Le Sciellour et al., 2019; Xiong et al., 2020), pushing it to dysbiosis. Dysbiosis (dysbacteriosis) is an alteration in the gut microbiota with an overgrowth of harmful microorganisms, or a depletion of beneficial bacteria, which can weaken the fragile equilibrium between the host and its GI microbiota (Walker, 2017; Ducatelle et al., 2018). A dysbiotic state is often associated with depression in nutrient digestion, loss of intestinal barrier function, and GI inflammation (Chen et al., 2015; Ducatelle et al., 2018), whereas eubiosis, referred to as a balanced microbial ecosystem (Iebba et al., 2016), can enhance health, productivity, and ability of chickens to withstand environmental stressors (Kogut, 2019). Although cutting-edge analytical techniques are currently available to study the GI microbiota (Borda-Molina et al., 2018), the modifications in structure, composition, and functions of the GI microbiota of heat-stressed chickens are still to be fully understood (He et al., 2021; Liu et al., 2022). However, changes in GI morphology, mucus quantity and composition, and attachment sites, coupled with an accumulation of poorly digested or even undigested feed components, are all plausible reasons for HS-caused dysbiosis.

The commensal microbiota is able to hinder the colonization and proliferation of allochthonous and pathogenic microorganisms in the GI ecosystem (Schneitz, 2005). This protective mechanism, conventionally termed competitive exclusion (CE) or “Nurmi concept”, was firstly observed in newly hatched chicks acquiring resistance to *Salmonella* challenges if previously inoculated *per os* with a suspension of crop and intestinal contents collected from healthy adult chickens (Nurmi and Rantala, 1973)*.* Chichlowski et al. (2007) specified that CE is a physical blockage of intestinal niches carried out by beneficial bacteria to the detriment of opportunistic pathogens. Desirable bacteria can also compete with pathogens for nutrients, and produce microbiostatic and microbicidal substances, such as organic acids and bacteriocins (Pan and Yu, 2014; Clavijo and Flórez, 2018). However, aberrant microbiotas of chickens subjected to HS have been related to an increased susceptibility to intestinal colonization of *Salmonella* Enteritidis (Burkholder et al., 2008; Soliman et al., 2009). Tsiouris et al. (2018) also demonstrated that HS can promote the expansion of *Clostridium perfringens* in the chicken intestine, becoming a predisposing factor for necrotic enteritis outbreak in flocks reared under hot conditions. *C*. *perfringens* can also release enterotoxins that, together with other harmful bacterial effectors, may impair TJs and gut barrier functions (Awad et al., 2017). Taken together, dysbiosis, intestinal barrier disorders, and mucosa inflammation are interconnected and fuel each other (Ducatelle et al., 2018), exacerbating the negative effects of HS on gut health, physiology, and performance of chickens (Tables 7, 8).

**TABLE 7:** Overview of heat stress effects on the GI microbiota of chickens.

Class	Heat stress effect	Pros	Cons	References^a^
GI microbiota	Perturbation of the GI ecosystem and microbial community stability → dysbiosis	—	Positive feedback loop among dysbiosis, GI barrier dysfunction, and GI inflammation → ↓ health and performance	**Suzuki et al. (1983)** **,** **Suzuki et al. (1989)**, **Lan et al. (2004)**, **Burkholder et al. (2008)**, **Soliman et al. (2009)**, **Song et al. (2014)**, Chen et al. (2018), **Tsiouris et al. (2018)**, **Wang X. J. et al. (2018)** **,** **Wang et al. (2020)**, **Ducatelle et al. (2018)**, **Shi et al. (2019)**, **Xing et al. (2019)**, **Zhu et al. (2019)**, **He et al.** (2019a), **He et al.** (**2019b)**, **He et al.** (2021), Le Sciellour et al. (2019), **Xiong et al. (2020)**, **Liu et al. (2020)** **,** **Liu et al. (2022**)
			↑ susceptibility to GI pathogen colonization → ↑ GI disorders (e.g., necrotic enteritis) → ↓ health and performance	

Upward arrow (↑), increase; downward arrow (↓), decrease; rightward arrow (→), consequence/degeneration.

a Include studies on non-avian species that have exhibited comparable heat stress effects and responses to those observed in chickens. Studies with the focus on chickens or poultry are highlighted in bold.

**TABLE 8:** Overview of heat stress effects on the inflammatory state of chickens.

Class	Heat stress effect	Pros	Cons	References^a^
Inflammatory state	Enteritis and systemic inflammation	Response to endotoxemia, microbial infection, and GI tissue injury	↑ energy expenditure to sustain the immune system → ↓ performance	**Quinteiro-Filho et al. (2010)** **,** **Quinteiro-Filho et al. (2012)** **,** Broom and Kogut (2018b), **Ruff et al. (2020)**

Upward arrow (↑), increase; downward arrow (↓), decrease; rightward arrow (→), consequence/degeneration.

a Include studies on non-avian species that have exhibited comparable heat stress effects and responses to those observed in chickens. Studies with the focus on chickens or poultry are highlighted in bold.

## Conclusion

Nowadays poultry farmers must deal with HS at almost every latitude because climate change has made high temperatures a pressing issue no longer limited to hot countries. Consequently, it would be advisable to update the estimate of costs and economic losses caused by HS to realize its actual impact on the global poultry industry.

**FIGURE 1:**
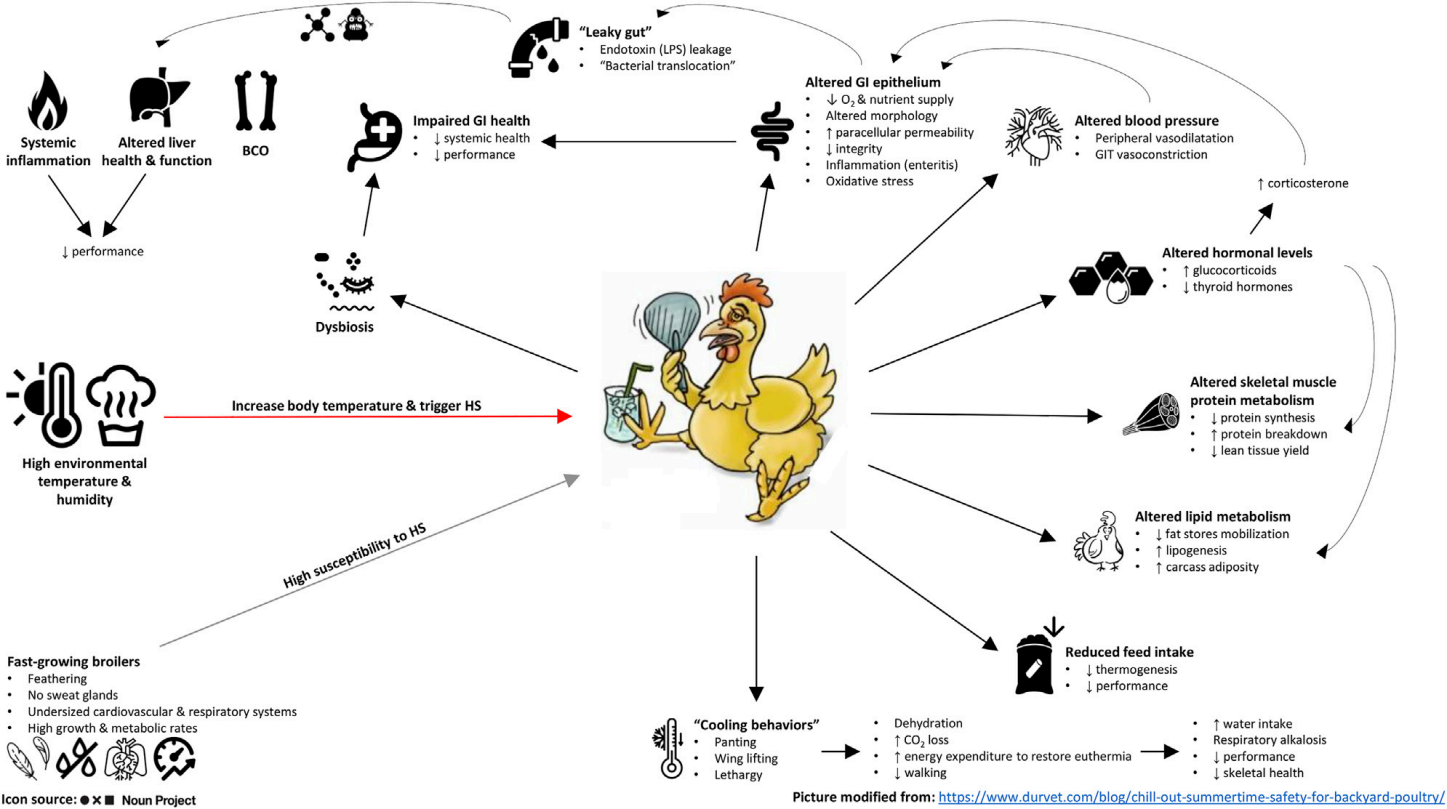
Summary chart of the main effects of heat stress on modern (broiler) chicken lines. LPS, lipopolysaccharides; BCO, bacterial chondronecrosis with osteomyelitis.

According to the literature reviewed here, HS provokes a wide range of deleterious effects on chickens, especially those belonging to modern high-performing lines (Figure 1). Firstly, HS negatively affects immunohomeostasis, hormonal equilibrium, and inflammatory and oxidative status. More studies on these physiologic alterations and their interconnections can help develop multitargeted solutions to help chickens combat HS more effectively. Secondly, HS promotes tissue catabolism and a substantial modification in protein and lipid metabolism. While there is evidence to assert that HS affects skeletal muscle accretion of chickens *via* both protein synthesis inhibition and protein degradation stimulation, further investigations are needed to clarify the underlying causes of the blunted fat mobilization in heat-stressed chickens. Lastly, high temperatures can be deemed to be a “dysbiogenic stressor” that undermines gut functionality and disrupts the host-microbiota interrelationship. Reinforcing the intestinal barrier, restoring digestive and absorptive processes, rebalancing the GI microbiota, and lowering the GI inflammation and oxidative stress seem therefore essential to increase HS tolerance and resilience for chickens.

In conclusion, reversing the homeostatic and metabolic perturbations induced by HS and conferring enteral protection appear to be promising approaches to fight against this growing threat to the poultry industry sustainability.

## Abbreviations

AA, amino acids; AMP/HDP, antimicrobial/host defense peptide; BCO, bacterial chondronecrosis with osteomyelitis; CE, competitive exclusion; FASN, fatty acid synthase; GI(T), gastrointestinal (tract); HS, heat stress; IFN-γ interferon gamma; IGF-1, insulin-like growth factor 1; IL-1β, interleukin 1 beta; LPS, lipopolysaccharides; MAFbx, muscle atrophy F-box; MLCK, myosin light-chain kinase; NEFA, non-esterified fatty acids; PAMP, pathogen-associated molecular pattern; PI3K, phosphatidylinositol 3-kinase; RNS, reactive nitrogen species; ROS, reactive oxygen species; S6K, p70S6 kinase; IgA, immunoglobulin A; T_3_, triiodothyronine; T_4_, thyroxine; TEER, transepithelial electrical resistance; TJ, tight junction; TLR, Toll-like receptor; TNF-α, tumor necrosis factor alpha; ZO, zonula occludens.
